# Use of Dietary Supplements in Patients Seeking Treatment at a Periodontal Clinic

**DOI:** 10.3390/nu5041110

**Published:** 2013-04-02

**Authors:** Bryan D. Johnston, Peter C. Fritz, Wendy E. Ward

**Affiliations:** 1 Faculty of Applied Health Sciences, Brock University, St. Catharines, Ontario, L2S 3A1, Canada; E-Mails: bb08ts@brocku.ca (B.D.J.); peter.fritz@utoronto.ca (P.C.F.); 2 Reconstructive Periodontics and Implant Surgery Clinic, Fonthill, Ontario, L0S 1E5, Canada

**Keywords:** dietary supplements, periodontal disease, supplement use, wound healing

## Abstract

Dietary supplement use may modify the risk of periodontal disease but effects on wound healing after periodontal procedures are less clear. This study characterized dietary supplement use by male and female patients (*n* = 376) attending a periodontal clinic—information that is essential for evidence-based intervention studies that may improve patient outcomes after periodontal procedures. Calcium, vitamin D, multivitamin and vitamin C were most commonly used. A greater (*p* ≤ 0.05) number of males took no supplements compared to females, and more (*p* ≤ 0.05) females than males took ≥ four supplements. Females took more (*p* ≤ 0.05) calcium, vitamin D, fish oil, green tea, magnesium, omega 3,6,9 and B vitamin complex. Younger patients (31–50 years) had the highest (*p* ≤ 0.05) frequency of no supplement use compared to older age groups. Patients over age 50 had a higher (*p* ≤ 0.05) frequency of using ≥ four supplements including calcium and vitamin D. Supplement use was lower (*p* ≤ 0.05) in smokers, particularly for calcium, fish oil, green tea and vitamin D. In conclusion, females, older individuals and non-smokers have higher supplement use. Future dietary intervention studies can focus on supplements with known biological activities—anti-inflammatory, antioxidant or osteogenic activity—that may enhance wound healing after reconstructive periodontal procedures.

## 1. Introduction

Periodontitis is the component of periodontal disease [1] associated with inflammation of the periodontium resulting in progressive bone and soft tissue destruction, ultimately leading to tooth loss [2]. The etiology of periodontitis originates with the development of a bacterial biofilm, or plaque, on the tooth surface and oral epithelia [2,3]. The host response to the pathogenic biofilm is to initiate periodontal inflammation and recruit polymorphonuclear neutrophils (PMNs) to the infection site [2,3]. PMNs respond to the microbial threats via the creation of reactive oxygen species (ROS), which in chronic inflammation, results in a state of oxidative stress [4].

Dietary supplement use may modify the risk for the development and progression of periodontal disease [5,6,7,8,9]. The antioxidant activity [4] of nutrients such as vitamin C [10,11], and α-tocopherol [12], and the anti-inflammatory activity [7] of polyunsaturated fatty acids (docosahexaeonic acid (DHA) [13,14]) may attenuate the development of periodontal disease. Studies using the NHANES III-a large cross-sectional survey study-have demonstrated that lower vitamin C intake is associated with a higher risk (OR 1.19) of having periodontal disease [10], and that higher vitamin C intake is associated with a reduced risk (OR 0.53) of severe periodontitis [11]. Additionally, using data from the NHANES III, the highest quintile of serum total antioxidant activity (TAOC) was associated with reduced risk (OR 0.63) of severe periodontitis [11]. Association studies have also shown that low intakes of α-tocopherol or DHA are associated with an increased risk of periodontal disease [12,13,14]. Large cross-sectional survey studies have identified that periodontal disease affects a substantial number of individuals and given the potential systemic health effects linked to periodontal disease (*i.e*., cardiovascular disease and preterm birth), periodontal disease represents a pubic health concern [3]. The Canadian Health Measures Survey from 2007 to 2009 reports that 21% of Canadian adults with natural teeth have experienced a moderate to severe periodontal problem [15], and current estimates from the United States (2009–2010 NHANES) report that periodontitis affects 47% of the adult population over 30 years of age [16]. At present, it is unclear exactly how much of the periodontal disease burden may be exacerbated by poor nutrition. Furthermore, there is limited data regarding whether dietary interventions may enhance recovery from periodontal disease. 

Physical disruption of the microbial biofilm at hygiene appointments is the first-line intervention toward periodontal disease [2]. However, emerging data from dietary intervention studies suggest a functional role for diet or dietary supplements in supporting periodontal health and attenuating periodontal disease [17,18,19]. For instance, a diet focusing on whole fresh foods—fruits, vegetables and whole-grains—while limiting processed foods, reduced periodontal inflammation in 20 women with metabolic syndrome [17]. Dietary intervention of two grapefruits per day for two weeks was associated with lower sulcus bleeding in patients with chronic periodontitis [18]. A diet rich in whole-grains has also been shown to reduce the risk of periodontitis by 23% in a prospective study of male health professionals [19]. Moreover, dietary supplements have been shown to have beneficial effects. Supplementation with a fruit and vegetable concentrate in capsules resulted in improvements in probing depth, bleeding on probing, and plaque scores [20]. Another supplementation study, using borage oil that contains high levels of omega-6, improved both probing depth and gingival inflammation in adults with periodontitis [21]. Supplementation with lycopene, in combination with oral prophylaxis, has also been shown to attenuate gingival inflammation more effectively than oral prophylaxis alone [22]. Together, the studies discussed highlight the potential importance of diet or dietary supplements in reducing periodontal inflammation in combination with regular hygiene maintenance.

In addition to maintenance of periodontal health, a few studies have shown that diet may assist with wound healing from periodontal procedures. These few studies have shown that micronutrients (vitamin D and the B vitamins) [23,24,25] and macronutrients (DHA and eicosapentaenoic acid (EPA)) [26,27] can improve patient recovery following periodontal therapy. Being vitamin D sufficient (serum 25(OH)D > 50 nmol/L) before open flap debridement surgery resulted in greater clinical attachment levels and reductions in probing depths post-surgery than patients with lower levels of serum 25(OH)D [23]. Likewise, patients receiving access flap surgery experienced better clinical attachment levels when treated post-operatively with a vitamin-B complex (50 mg thiamine HCl, riboflavin, niacinamide, d-calcium pantothenate, pyridoxine HCl; 50 μg d-biotin, cyanocobalamin; 400 μg folate) [24]. Vitamin B12 as a component of post-surgical medication also resulted in less patient pain at 6 and 120 h post third molar extraction than in control patients [25]. In patients requiring sanative therapy, a combination of acetylsalicylic acid (81 mg) and fish oil (containing 900 mg DHA and EPA) decreased probing depths while increasing clinical attachment and reducing levels of salivary RANKL and MMP-8, markers of inflammation [26]. Similarly, in patients with a furcation defect requiring bone allograft, a combination therapy of acetylsalicylic acid (75 mg) and DHA (900 mg) and EPA (450 mg) resulted in greater clinical attachment, probing depth reductions, and decreased amounts of IL-1β present in the gingival crevicular fluid [27].

Together, findings from these studies suggest that use of dietary supplements during and after periodontal procedures may improve periodontal health outcomes following periodontal procedures. However, randomized controlled trials are needed to more definitively determine how dietary supplements may enhance outcomes after periodontal procedures. The objective of this study was to characterize the use of dietary supplements by patients who attend a periodontal clinic for one of three reasons: comprehensive general examination, implant consultation or other surgical consultation. By understanding the pattern of intakes, researchers will be better informed to design intervention studies that will support better long-term outcomes after periodontal procedures.

## 2. Methods

A sample of 442 surveys was collected from a periodontal clinic in Southern Ontario. The surveys were administered as part of the routine patient information collected before a periodontal procedure was performed. Patients with uncommon reasons for visiting the periodontal clinic and those in the 19–30 years of age category were removed from the dataset due to small sample group sizes. The final dataset contained 376 surveys with the following three most frequent ‘reason for visit’ categories: Comprehensive General Examination (*n* = 90), Implant Consultation (*n* = 126), and Other Surgical Consultation (*n* = 160). The surgical consult group contained the following procedures: crown lengthening, flap surgery, and grafting. A supplement was considered used if the patient indicated any use of the supplement, irrespective of brand, dose, frequency, or duration. The frequency of supplement use among groups was assessed using a Chi-square test with significance defined as *p* ≤ 0.05. This study was approved by the Human Ethics Board at Brock University, St. Catharines, Ontario (File #11-161-Ward). 

## 3. Results

### 3.1. Characteristics of the Study Population

The study population of 376 patients consisted of 152 males (40.4%) and 224 females (59.6%). Approximately 57% of patients were between the ages of 51 and 70 years and most were receiving a surgical consultation (42.6%). A smoking frequency of 16.0% was reported for males and females combined (Table 1).

**Table 1:** Characteristics of Study Population.

Characteristic		Male	Female	Total
		*n* = 152	*n* = 224	*n* =376
		*n* (%)	*n* (%)	*n* (%)
Age				
	31–50 years	45 (29.6)	70 (31.2)	115 (30.6)
	51–70 years	85 (55.9)	131 (58.5)	216 (57.5)
	≥70 years	22 (14.5)	23 (10.3)	45 (12.0)
Smoking Status				
	Ever Smoker	28 (18.4)	32 (14.3)	60 (16.0)
	Never Smoker	124 (81.6)	192 (85.7)	316 (84.0)
Reason for Visit				
	Comprehensive General Examination	37 (24.3)	53 (23.7)	90 (23.9)
	Implant Consultation	56 (36.8)	70 (31.2)	126 (33.5)
	Other Surgical Consultation	59 (38.8)	101 (45.1)	160 (42.6)

### 3.2. Number of Supplements Used According to Sex, Age and Smoking Status

There were a greater (*p* ≤ 0.05) number of males taking no supplements compared to females. Likewise, there were more (*p* ≤ 0.05) females taking ≥ four supplements than males (Table 2). Taking no supplements depended on the participant age. The youngest age group (31–50 years) had the highest (*p* ≤ 0.05) frequency of no supplement use compared to the 51–70 years group, which was in turn higher (*p* ≤ 0.05) than the ≥70 years of age group (Table 2). The 30–51 years group had a higher (*p* ≤ 0.05) frequency of using one supplement compared to the 51–70 years group but not the ≥70 years of age group (Table 2). Complementary to the pattern of younger participants not using supplements, the older age groups (≥70 years and 51–70 years) had a higher (*p* ≤ 0.05) frequency of using four or more supplements (Table 2). Supplement use was lower (*p* ≤ 0.05) in smokers than non-smokers (Table 2).

**Table 2:** Total Supplements Used by Sex, Age and Smoking Status.

Total Supplements	Male	Female	31-50 years	51-70 years	≥ 70 years	Current Smoker	Non-Smoker
	*n* = 152	*n* = 224	*n* = 115	*n* = 216	*n* = 45	*n* = 60	*n* = 316
	*n* (%)	*n* (%)	*n* (%)	*n* (%)	*n* (%)	*n* (%)	*n* (%)
0	70 (46.1) ^a^	65 (29.0) ^b^	55 (47.8) ^a^	73 (33.8) ^b^	7 (15.6) ^c^	35 (58.3) ^a^	100 (31.6) ^b^
1	28 (18.4)	36 (16.1)	26 (22.6) ^a^	29 (13.4) ^b^	9 (20.0) ^a,b^	6 (10.0)	58 (18.4)
2	14 (9.2)	30 (13.4)	11 (9.6)	26 (12.0)	7 (15.6)	5 (8.3)	39 (12.3)
3	14 (9.2)	29 (12.9)	11 (9.6)	27 (12.5)	5 (11.1)	5 (8.3)	38 (12.0)
≥4	26 (17.1) ^b^	64 (28.6) ^a^	12 (10.4) ^b^	61 (28.2) ^a^	17 (37.8) ^a^	9 (15.0)	81 (25.6)

Within sex, age, and smoking status, different superscripts in a row denote significant differences among groups (*p* ≤ 0.05).

### 3.3. 10 Most Frequently Used Supplements by Total Population and by Sex

The 10 most frequently used supplements by the entire population were vitamin D, multivitamin, calcium, vitamin C, omega 3,6,9, vitamin B12, B vitamin complex, fish oil, magnesium, and green tea (Table 3). Although not in the same order, both males and females reported the same four supplements as those most frequently used (calcium, multivitamin, vitamin C, vitamin D). Omega 3,6,9 was ranked the fifth most used supplement for both males and females. Glucosamine, ground flaxseed, and garlic were unique to the 10 most frequently used supplements in the male population. The female population reported the same 10 most frequently used supplements as the general population, but not in the same ranked order (Table 3). Specifically, females reported greater frequency of use for B vitamin complex (*p* = 0.020), calcium (*p* ≤ 0.001), fish oil (*p* = 0.041), green tea (*p* = 0.003), magnesium (*p* = 0.001), omega 3,6,9 (*p* = 0.042), and vitamin D (*p* = 0.001) compared to males (Table 4).

**Table 3:** 10 Most Frequently Used Supplements.

Supplement Name	Total Population	Supplement Name	Male	Supplement Name	Female
	*n* = 376		*n* = 152		
					*n* = 224
			*n* (%)		*n* (%)
	*n* (%)				
Vitamin D	117 (31.1)	Multivitamin	45 (29.6)	Vitamin D	84 (37.5)
Multivitamin	117 (31.1)	Vitamin D	33 (21.7)	Calcium	83 (37.1)
Calcium	97 (25.8)	Vitamin C	22 (14.5)	Multivitamin	72 (32.1)
Vitamin C	61 (16.2)	Calcium	14 (9.2)	Vitamin C	39 (17.4)
Omega 3,6,9	49 (13.0)	Omega 3,6,9	13 (8.6)	Omega 3,6,9	36 (16.1)
Vitamin B12	45 (12.0)	Vitamin B12	13 (8.6)	B Vitamin Complex	32 (14.3)
B Vitamin Complex	42 (11.2)	Glucosamine	11 (7.2)	Vitamin B12	32 (14.3)
Fish Oil	40 (10.6)	B Vitamin Complex	10 (6.6)	Magnesium	31 (13.8)
Magnesium	37 (9.8)	Fish Oil	10 (6.6)	Fish Oil	30 (13.4)
Green Tea	35 (9.3)	Flaxseed Ground *	6 (3.9)	Green Tea	29 (12.9)
		Garlic *	6 (3.9)		
		Green Tea *	6 (3.9)		
		Magnesium *	6 (3.9)		

* Supplements with same frequency of use in 10th position.

**Table 4:** Sex Differences in Supplements Used.

Supplement Name	Male, *n* = 152, *n* (%)	Female, *n* = 224, *n* (%)	*p* value
B Vitamin Complex	10 (6.6) ^b^	32 (14.3) ^a^	0.020
Calcium	14 (9.2) ^b^	83 (37.1) ^a^	<0.001
Fish Oil	10 (6.6) ^b^	30 (13.4) ^a^	0.041
Green Tea	6 (3.9) ^b^	29 (12.9) ^a^	0.003
Magnesium	6 (3.9) ^b^	31 (13.8) ^a^	0.001
Omega 3,6,9	13 (8.6) ^b^	36 (16.1) ^a^	0.042
Vitamin D	33 (21.7) ^b^	84 (37.5) ^a^	0.001

Different superscripts in a row denote significant differences among groups (*p* ≤ 0.05); Use of other supplements did not significantly differ by sex.

### 3.4. 10 Most Frequently Used Supplements by Age

Calcium, multivitamin, vitamin C, and vitamin D were the four most frequently used supplements across the age categories, although they were not in the same order for each category, except vitamin C in the forth ranked position (Table 5). In the 31–50 years group, a multivitamin was the most frequently used supplement followed by vitamin D and calcium. In the 51–70 years group, multivitamin was replaced by vitamin D as the most used frequently used supplement and calcium use remained the same in the third position. In the oldest age group, ≥70 years, a multivitamin was replaced by calcium as the second most used supplement while vitamin D remained the most used supplement. Unlike the 51–70 and ≥70 years of age categories, the 31–50 years of age category did not include magnesium. Similarly, the 51–70 years of age group, unlike the other age categories, did not include ground flaxseed. The ≥70 years of age group was unique in listing glucosamine as one of its 10 most frequently used supplements (Table 5). Both calcium (*p* = 0.001) and vitamin D (*p* ≤ 0.001) use were dependent on the age of the study participant, as the older age categories ≥70 years and 51–70 years reported a higher usage frequency than the 31–50 years of age group (Table 6).

**Table 5:** 10Most Frequently Used Supplements by Age.

Supplement Name	31–50 years	Supplement Name	51–70 years	Supplement Name	≥70 years
	*n* = 115		*n* = 216		*n* = 45
	*n* (%)		*n* (%)		*n* (%)
Multivitamin	32 (27.8)	Vitamin D	77 (35.6)	Vitamin D	20 (44.4)
Vitamin D	20 (17.4)	Multivitamin	73 (33.8)	Calcium	18 (40.0)
Calcium	16 (13.9)	Calcium	63 (29.2)	Multivitamin	12 (26.7)
Vitamin C	15 (13.0)	Vitamin C	35 (16.2)	Vitamin C	11 (24.4)
B Vitamin Complex	10 (8.7)	Omega 3,6,9	30 (13.9)	Magnesium	9 (20.0)
Fish Oil	10 (8.7)	Vitamin B12	30 (13.9)	Omega 3,6,9	9 (20.0)
Omega 3,6,9	10 (8.7)	B Vitamin Complex	28 (13.0)	Fish Oil	7 (15.6)
Green Tea	8 (7.0)	Magnesium	24 (11.1)	Vitamin B12	7 (15.6)
Vitamin B12	8 (7.0)	Fish Oil	23 (10.6)	Glucosamine	6 (13.3)
Flaxseed Ground	5 (4.3)	Green Tea	23 (10.6)	B Vitamin Complex *	4 (8.9)
				Flaxseed Ground *	4 (8.9)
				Green Tea *	4 (8.9)

* Supplements with same frequency of use in 10th position.

**Table 6:** Age and Supplements Used.

Supplement Name	31–50 years	51–70 years	≥70 years	*p* value
	*n* = 115	*n* = 216	*n* = 45	
	*n* (%)	*n* (%)	*n* (%)	
Calcium	16 (13.9) ^b^	63 (29.2) ^a^	18 (40.0) ^a^	0.001
Vitamin D	20 (17.4) ^b^	77 (35.6) ^a^	20 (44.4) ^a^	<0.001

Different superscripts in a row denote significant differences among groups (*p* ≤ 0.05); Use of other supplements did not significantly differ by age.

### 3.5. 10 Most Frequently Used Supplements by Smoking Status

Current smokers reported the same four most frequently used supplements (calcium, multivitamin, vitamin C, and vitamin D) as non-smokers (Table 7). Both the current smoker and non-smoker populations reported using omega 3,6,9 as the fifth most used supplement, as well and both vitamin B12 and B vitamin complex as the seventh and eighth most used supplements. Unlike the current smokers, the non-smokers reported using fish oil and green tea while current smokers reported using cod liver oil and garlic in their 10 most frequently used supplements. Despite the differences in the ranking of the 10 most frequently used supplements, the usage frequencies for supplements were not the same. Current smokers reported using less calcium (*p* = 0.006), fish oil (*p* = 0.011), green tea (*p* = 0.027) and vitamin D (*p* = 0.022) than non-smokers (Table 8).

**Table 7:** 10 Most Frequently Used Supplements By Smoking Status.

Supplement Name	Current Smoker	Supplement Name	Non-Smoker
	*n* = 60		
			*n* = 316
	*n* (%)		*n* (%)
Multivitamin	14 (23.3)	Vitamin D	106 (33.5)
Vitamin D	11 (18.3)	Multivitamin	103 (32.6)
Vitamin C	9 (15.0)	Calcium	90 (28.5)
Calcium	7 (11.7)	Vitamin C	52 (16.5)
Omega 3,6,9	7 (11.7)	Omega 3,6,9	42 (13.3)
Magnesium	6 (10.0)	Fish Oil	39 (12.3)
Vitamin B12	6 (10.0)	Vitamin B12	39 (12.3)
B Vitamin Complex	4 (6.7)	B Vitamin Complex	38 (12.0)
Cod Liver Oil	3 (5.0)	Green Tea	34 (10.8)
Garlic	3 (5.0)	Magnesium	31 (9.8)

**Table 8:** Smoking and Supplements Used.

Supplement Name	Current Smoker, *n* = 60, *n* (%)	Non-Smoker, *n* = 316, *n* (%)	*p* value
Calcium	7 (11.7) ^b^	90 (28.5) ^a^	0.006
Fish Oil	1 (1.7) ^b^	39 (12.3) ^a^	0.011
Green Tea	1 (1.7) ^b^	34 (10.8) ^a^	0.027
Vitamin D	11 (18.3) ^b^	106 (33.5) ^a^	0.022

Different superscripts in a row denote significant differences among groups (*p* ≤ 0.05); Use of other supplements did not significantly differ between current and non-smokers.

### 3.6. 10 Most Frequently Used Supplements by Reason for Visit

For the three reasons for visit, the 10 most frequently used supplements were similar and included calcium, multivitamin and vitamin D (Table 9). Only the comprehensive general examination group reported using glucosamine as one of their 10 most frequently used supplements. Similarly, the implant consultation group was unique in reporting ground flaxseed as one of its 10 most frequently used supplements (Table 9). Patients receiving a surgical consultation had a lower (*p* = 0.009) usage frequency for glucosamine than either the comprehensive general examination or implant consultation categories. 

**Table 9:** 10 Most Frequently Used Supplements by Reason for Visit.

Supplement Name	Comprehensive General Examination	Supplement Name	Implant Consultation	Supplement Name	Other Surgical Consultation
	*n* = 90		*n* = 126		*n* = 160
	*n* (%)		*n* (%)		*n* (%)
Multivitamin	30 (33.3)	Vitamin D	43 (34.1)	Vitamin D	49 (30.6)
Vitamin D	25 (27.8)	Multivitamin	42 (33.3)	Calcium	45 (28.1)
Calcium	24 (26.7)	Calcium	28 (22.2)	Multivitamin	45 (28.1)
Omega 3,6,9	15 (16.7)	Omega 3,6,9	19 (15.1)	Vitamin C	29 (18.1)
Vitamin C	13 (14.4)	Vitamin C	19 (15.1)	B Vitamin Complex	21 (13.1)
B Vitamin Complex	12 (13.3)	Fish Oil	17 (13.5)	Vitamin B12	21 (13.1)
Glucosamine	12 (13.3)	Vitamin B12	16 (12.7)	Magnesium	17 (10.6)
Green Tea	9 (10.0)	Green Tea	12 (9.5)	Fish Oil	15 (9.4)
Fish Oil	8 (8.9)	Magnesium	12 (9.5)	Omega 3,6,9	15 (9.4)
Magnesium *	8 (8.9)	Flaxseed Ground	10 (7.9)	Green Tea	14 (8.8)
Vitamin B12 *	8 (8.9)				

* Supplements with same frequency of use in 10th position.

## 4. Discussion

This study showed that females used more supplements than males, and that general supplement use increased with subject age, regardless of sex. Moreover, smokers used fewer supplements than non-smokers. Interestingly, the list of the 10 most commonly used dietary supplements was relatively similar when comparing by sex, age, smoking status or reason for visit. 

The finding that females used more supplements than males, and that supplement use increased with subject age, was not surprising based on data from the Canadian Community Health Survey (2004–2005). The Canadian Community Health Survey (CCHS) cycle 2.2 is a nationwide cross-sectional nutrition survey representing 98% of all province-dwelling Canadians (*n* = 35,107). The CCHS cycle 2.2 had a response rate of 76.5% and as part of its design included an assessment of the nutritional supplements used by each participant [28]. According to the CCHS 2.2, total supplement use by Canadians was 40.1% [29] and multivitamin use, defined as ≥ three supplements, was 28% [30]. Total supplement use in the present study was 64.1%, much greater than the CCHS but may be explained by the fact that patients visiting the periodontal clinic had a higher proportion of older individuals in which supplement use is higher. Another reason for the difference may be the fact that we used a detailed list of supplements whereas the CCHS focused on multivitamin use without consideration of herbals or botanicals [28]. Similar to our findings, Shakur *et al*., 2012 reported greater supplement use between the NHANES survey and the CCHS 2.2, with the difference being attributed to the fact that NHANES examines a greater variety of supplements (such as herbals or botanicals) than the CCHS 2.2 [30]. The fact that our survey contained a wide variety of possible nutritional supplements, not limited to vitamins and minerals, may explain our greater percentage of total supplement use compared to the CCHS 2.2. However, good agreement between the studies was observed for individual supplement use such as multivitamin (28.0% *vs*. 31.1%), vitamin D (28.0% *vs*. 31.1%), and calcium (28.0% *vs*. 25.8%), in which the first percentage is from the CCHS 2.2 [30]. Additionally, the CCHS 2.2 and NHANES (2003–2006) studies, similar to the findings of the present study, reported greater supplement use among women compared to men, and greater supplement use with older age [29,30,31] .

Given the challenge of attaining the dietary reference intake for calcium and vitamin D from diet alone in the Canadian elderly [32,33,34,35,36] and public health messages encouraging use of vitamin D supplements, it was not surprising that calcium and vitamin D supplement use increased in the post-50 age categories. As part of the Canada’s Food Guide, Health Canada advises that all Canadians 50 years or older consume a vitamin D supplement containing 400 IU per day [37]. Moreover, other organizations such as the Canadian Cancer Society suggest Canadians consume a vitamin D supplement containing 1000 IU in the winter months [38]. In addition to calcium and vitamin D, multivitamin and vitamin C supplement use were also included in the four most used supplements after age 50 years. This is supported by a survey of Canadian community-living older adults in which calcium, vitamin C and D were frequently used micronutrients and overall multivitamin use was high (43.5%) [35]. In a study of Ontario seniors living in long term care facilities, supplementation with the recommended vitamin D dose (>400 IU/day) was effective in ensuring that >90% of the seniors achieved optimum serum 25(OH)D levels (>75 nmol/L) [36].

Vitamin C is critical for the maintenance of periodontal health. It is well known that smoking depletes vitamin C stores and thus smokers should consume a vitamin C supplement or increase their dietary intake of vitamin C [39]. However, use of vitamin C supplements was not higher among smokers in this study. This finding is similar to findings from the CCHS 2.2 that reported no difference in vitamin C supplement use between smokers and non-smokers [39]. Whether supplementation with vitamin C improves wound healing after periodontal surgery in smokers requires investigation. Our study also showed that current smokers used less calcium, vitamin D, green tea and fish oil supplements. The effect of these supplements in smokers *versus* non-smokers at improving wound healing after periodontal procedures is an area for future investigation. 

Supplement use was largely similar among patients regardless of their reason for visit. The only difference was that patients receiving a surgical consultation had lower usage frequency of glucosamine than patients receiving comprehensive general examination or implant consultation. There is no clear link between glucosamine supplement use and the different reasons for visiting the periodontist. However, males and those over 70 years of age did report glucosamine as one of the most commonly used supplements and may have been more represented in the comprehensive general examination or implant consultation groups.

## 5. Conclusions

Supplement usage in this sample of patients seeking periodontal care was similar to the general Canadian population and was not dependent on the patient’s reason for visiting the periodontist. Future dietary intervention studies to optimize periodontal health can focus on supplements with known biological activities that may enhance wound healing after periodontal procedures. Dietary supplements such as those with known anti-inflammatory, antioxidant or osteogenic activity are of interest.
